# Lifetime and age-conditional risk estimates of end-stage kidney disease requiring maintenance dialysis in Japan

**DOI:** 10.1007/s10157-020-01860-5

**Published:** 2020-02-10

**Authors:** Minako Wakasugi, Ichiei Narita

**Affiliations:** 1 Division of Comprehensive Geriatrics in Community, Niigata University Graduate School of Medical and Dental Sciences, 1-757 Asahimachi, Chuo-ku, Niigata, 951-8510 Japan; 2 Division of Clinical Nephrology and Rheumatology, Niigata University Graduate School of Medical and Dental Sciences, Niigata, Japan

**Keywords:** Age-conditional risk, Lifetime risk, Epidemiology, Public health, Mortality

## Abstract

**Background:**

Lifetime risk is an epidemiologic measure that expresses the probability of disease in the remaining lifetime for an index age. It is also an easily understandable statistical measure used to communicate the absolute risk of disease to the lay population. The lifetime risk of end-stage kidney disease (ESKD) has never been reported for the Japanese population. Here, we used data from the Japanese Society of Dialysis Therapy (JSDT) to estimate the lifetime risk of ESKD by sex in Japan.

**Methods:**

The lifetime risk of ESKD was estimated using life-table methods. We defined an incident case of ESKD as a patient with loss of kidney function that resulted in maintenance dialysis therapy. The number of incident cases of ESKD and number of ESKD deaths in 2017 were obtained from data published by the JSDT. The population and total number of deaths in Japan for the same year were obtained from National Vital Statistics. By including all-cause mortality, risks were adjusted for competing causes of death.

**Results:**

The cumulative incidence of ESKD from birth until age 95 years was 3.14% [95% confidence interval (CI) 3.10–3.18] for men and 1.42% (1.39–1.44) for women. These probabilities illustrate that approximately 1 in 32 men and 1 in 71 women in Japan will develop ESKD that results in maintenance dialysis therapy in their lifetime.

**Conclusions:**

Considerable sex differences were found in the lifetime risk of ESKD in Japan. This easily understandable information could be used to assist in public health education and planning.

## Introduction

Easily understandable statistics are essential for communicating the absolute risk of end-stage kidney disease (ESKD) to the lay population, policy makers, and health service providers, and for predicting the disease burden of ESKD. We previously reported the age-standardized incidence [1] and mortality rates [2–5] of ESKD. An alternative measure is lifetime risk, an epidemiologic measure that expresses the probability of disease in the remaining lifetime for an index age. Lifetime risk is often the preferred measure used in public health communications, as it is perceived to be both informative and easily understandable.

To date, no studies have reported on the lifetime risk of ESKD in the Japanese population. Using data from the Japanese Society of Dialysis Therapy (JSDT), we estimated the lifetime risk of ESKD by sex in Japan.

## Materials and methods

### Data source

An incident case of ESKD was defined as a patient with loss of kidney function that resulted in maintenance dialysis therapy including both hemodialysis and peritoneal dialysis. Because the number of pre-emptive kidney transplant patients is small in Japan, with only 101 patients being reported in 2009 [6], this definition covers almost all ESKD patients for whom renal replacement therapy is initiated in Japan. The number of incident cases of ESKD and number of deaths among patients with ESKD in 2017 were extracted from an annual data report of the JSDT [7]. In brief, the JSDT registry collects data every year by sending questionnaires to all dialysis facilities in Japan. This registry collected information on the epidemiological features, treatment, and outcome of individual dialysis patients with a very high response rate of 98.8% in 2017. Details on JSDT registry data collection techniques are available online at the JSDT website (https://www.jsdt.or.jp/english/2426.html). The population and total number of deaths in Japan for the same year were obtained from National Vital Statistics provided by the Ministry of Health, Labour and Welfare (https://www.e-stat.go.jp/SG1/estat/eStatTopPortalE.do).

This study was conducted according to the principles of the Declaration of Helsinki, Japanese privacy protection laws, and Ethical Guidelines for Medical and Health Research Involving Human Subjects published by the Ministry of Education, Science and Culture, and the Ministry of Health, Labour and Welfare in 2015. The current analyses used existing national data without any individual patient data. The Institutional Review Board (IRB) of our university did not require IRB review for this study, because secondary analysis using existing figures without any individual patient identifiers is not considered research involving human subjects and is thus exempt from IRB review.

### Statistical analysis

Data were categorized into 5-year age groups, with 95 years and older being an open-ended group. The incidence rate for each 5-year age interval was estimated by dividing the number of incident ESKD cases in each period by the size of the population in that period [1, 8]. Mortality rates for each year were estimated similarly to incidence rates [2–5]. The number of deaths prior to ESKD onset (competing events) was determined by subtracting the number of deaths among patients with ESKD for each period from the total number of deaths for the period.

Risks (probabilities) of being diagnosed with ESKD during a given age interval and the cumulative incidence of ESKD for 2017 from birth until age 95 for both sexes were estimated using the life-table methods of Fay [9] and Fay et al. [10] with DevCan software (version 6.7.4) developed at the National Cancer Institute [11]. Briefly, age-specific mortality and incidence rates were applied to a hypothetical cohort of 10 million live births. By including all-cause mortality, risks were adjusted for competing causes of death. The underlying assumption of all calculated risks was that the incidence and mortality rates were constant during the time window for which the risk was estimated. This version of DevCan splits the 0–4 age group into two groups, i.e., 0-year-olds are in a group separate from the 1–4-year-old group. Our data included the population and total number of deaths in Japan for the 0-year-old and 1–4-year-old groups. Unfortunately, however, only the number of incident cases of ESKD and number of ESKD deaths for the 0–4 age group were available in this study. As both the number of incident cases of ESKD and number of ESKD deaths for the 0–4 age group were very small, we assumed the number of incident cases of ESKD and number of ESKD deaths for the 0-year-old group to be zero in this study.

## Results

Table 1 shows a composite life table by sex, which includes the cumulative probability of developing ESKD from birth and cumulative probability of dying without ESKD from birth. In this hypothetical population of 10 million live births, 968,194 men and 2,511,379 women live to be over age 95 years. The numbers of people who develop ESKD in this hypothetical cohort peak in the 75–79 age group in men and in the 80–84 age group in women. With the exception of a relatively large number of infant deaths, the number of deaths due to other causes rises through the 85–89 age group and then falls in men, whereas the number rises with age in women.

**Table 1:** Probability of developing ESKD in Japan

Age interval	Male					Female				
	Total alive and at risk at beginning of interval	Number of individuals who develop ESKD in this interval	Number of individuals who die from other causes in this interval	Cumulative probability of developing ESKD from birth (%)	Cumulative probability of dying without ESKD from birth (%)	Total alive and at risk at beginning of interval	Number of individuals who develop ESKD in this interval	Number of individuals who die from other causes in this interval	Cumulative probability of developing ESKD from birth (%)	Cumulative probability of dying without ESKD from birth (%)
0–4	10,000,000	131	38,344	0.00	0.38	10,000,000	118	35,878	0.00	0.36
5–9	9,961,525	94	4,709	0.00	0.43	9,964,004	57	3,565	0.00	0.39
10–14	9,956,722	85	5,816	0.00	0.49	9,960,382	106	3,304	0.00	0.43
15–19	9,950,821	237	13,231	0.01	0.62	9,956,972	162	5,987	0.00	0.49
20–24	9,937,352	753	21,616	0.01	0.84	9,950,823	298	9,042	0.01	0.58
25–29	9,914,984	1,381	24,298	0.03	1.08	9,941,484	652	11,898	0.01	0.70
30–34	9,889,305	2,594	29,570	0.05	1.38	9,928,935	1,093	15,720	0.02	0.85
35–39	9,857,141	4,367	38,691	0.10	1.76	9,912,122	1,733	22,161	0.04	1.08
40–44	9,814,082	7,398	57,390	0.17	2.34	9,888,229	2,690	35,275	0.07	1.43
45–49	9,749,294	12,436	91,005	0.29	3.25	9,850,264	4,477	53,717	0.11	1.97
50–54	9,645,852	18,269	143,843	0.48	4.69	9,792,070	6,394	79,883	0.18	2.76
55–59	9,483,741	22,683	227,909	0.70	6.96	9,705,794	8,438	113,799	0.26	3.90
60–64	9,233,148	28,893	362,599	0.99	10.59	9,583,557	11,279	165,513	0.37	5.56
65–69	8,841,657	35,796	557,655	1.35	16.17	9,406,765	14,507	251,564	0.52	8.07
70–74	8,248,206	43,709	793,389	1.79	24.10	9,140,694	17,823	384,150	0.70	11.91
75–79	7,411,108	47,691	1,143,267	2.27	35.53	8,738,721	21,868	659,227	0.92	18.51
80–84	6,220,150	45,311	1,633,315	2.72	51.87	8,057,626	24,399	1,191,048	1.16	30.42
85–89	4,541,523	29,723	1,932,589	3.02	71.19	6,842,180	17,215	1,916,242	1.33	49.58
90–94	2,579,212	10,391	1,600,627	3.12	87.20	4,908,723	6,940	2,390,404	1.40	73.48
≥ 95	968,194	1,675	966,519	3.14	96.86	2,511,379	1,548	2,509,831	1.42	98.58

*ESKD* end-stage kidney disease

**Fig. 1:**
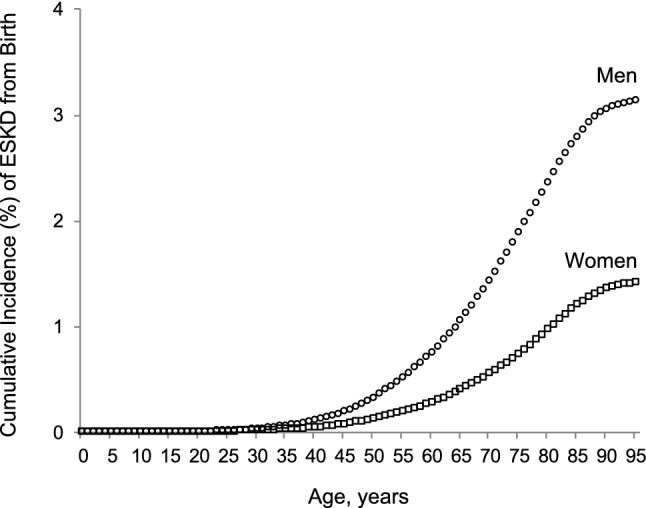
Cumulative incidence (%) of end-stage kidney disease (ESKD) from birth to age 95 years by sex in Japan in 2017. Open circles and squares represent cumulative incidences for males and females, respectively

The cumulative incidence of ESKD from birth begins to rise at approximately age 40 years and nearly plateaus around age 90 years in both sexes (Fig. 1). The cumulative lifetime incidence of ESKD from birth was 3.14% [95% confidence interval (CI) 3.10–3.18%] for men and 1.42% (95% CI 1.39–1.44%) for women. These probabilities illustrate that approximately 1 in 32 men and 1 in 71 women in Japan will develop ESKD that results in maintenance dialysis therapy during their lifetime.

Tables 2 and 3 show age-conditional probabilities of developing ESKD by sex. ‘Eventually’ denotes the residual lifetime probability of those who have not developed ESKD at ‘Current age.’ For example, a male who has not developed ESKD at age 60 years has a 1.69% (95% CI 1.66–1.72) chance of developing ESKD before age 80 years, and a 2.63% (95% CI 2.60–2.67)—or 1 in 38 (95% CI 37–38)—chance of developing ESKD during his lifetime.

**Table 2:** Percentage of Japanese men who develop ESKD by specified age

Current age	Develop ESKD by specified age									Eventually^a^	1 in:
	10 years	20 years	30 years	40 years	50 years	60 years	70 years	80 years	90 years		
0 year	0.002 (0.001, 0.004)	0.005 (0.004, 0.008)	0.03 (0.02, 0.03)	0.10 (0.09, 0.10)	0.29 (0.28, 0.31)	0.70 (0.69, 0.72)	1.35 (1.33, 1.37)	2.27 (2.23, 2.30)	3.02 (2.98, 3.05)	3.14 (3.10, 3.18)	32 (31, 32)
10 years		0.003 (0.002, 0.005)	0.02 (0.02, 0.03)	0.09 (0.09, 0.10)	0.29 (0.28, 0.30)	0.71 (0.69, 0.72)	1.35 (1.33, 1.38)	2.27 (2.24, 2.30)	3.03 (2.99, 3.06)	3.15 (3.11, 3.19)	32 (31, 32)
20 years			0.02 (0.02, 0.02)	0.09 (0.09, 0.10)	0.29 (0.28, 0.30)	0.70 (0.69, 0.72)	1.35 (1.33, 1.38)	2.27 (2.24, 2.30)	3.03 (2.99, 3.07)	3.15 (3.11, 3.19)	32 (31, 32)
30 years				0.07 (0.07, 0.08)	0.27 (0.26, 0.28)	0.69 (0.67, 0.70)	1.34 (1.32, 1.36)	2.26 (2.23, 2.29)	3.02 (2.99, 3.06)	3.14 (3.11, 3.18)	32 (31, 32)
40 years					0.20 (0.19, 0.21)	0.62 (0.60, 0.64)	1.28 (1.26, 1.30)	2.21 (2.18, 2.24)	2.97 (2.94, 3.01)	3.10 (3.06, 3.14)	32 (32, 33)
50 years						0.42 (0.41, 0.44)	1.10 (1.07, 1.12)	2.04 (2.01, 2.07)	2.82 (2.78, 2.86)	2.95 (2.91, 2.98)	34 (34, 34)
60 years							0.70 (0.69, 0.72)	1.69 (1.66, 1.72)	2.50 (2.47, 2.54)	2.63 (2.60, 2.67)	38 (37, 38)
70 years								1.11 (1.09, 1.13)	2.02 (1.99, 2.05)	2.16 (2.13, 2.20)	46 (45, 47)
80 years									1.21 (1.18, 1.24)	1.40 (1.37, 1.44)	71 (70, 73)
90 years										0.47 (0.43, 0.50)	214 (198, 230)

Values are presented as incidence (95% confidence interval) in percentages, except for the column “1 in:,” which shows numbers (95% confidence interval)

*ESKD* end-stage kidney disease

^a^“Eventually” corresponds to follow-up of more than 95 years

**Table 3:** Percentage of Japanese women who develop ESKD by specified age

Current age	Develop ESKD by specified age									Eventually^a^	1 in:
	10 years	20 years	30 years	40 years	50 years	60 years	70 years	80 years	90 years		
0 year	0.002 (0.001, 0.004)	0.004 (0.003, 0.007)	0.01 (0.01, 0.02)	0.04 (0.04, 0.05)	0.11 (0.11, 0.12)	0.26 (0.25, 0.27)	0.52 (0.51, 0.54)	0.92 (0.90, 0.94)	1.33 (1.31, 1.36)	1.42 (1.39, 1.44)	71 (69, 72)
10 years		0.003 (0.002, 0.004)	0.01 (0.01, 0.02)	0.04 (0.04, 0.05)	0.11 (0.11, 0.12)	0.26 (0.25, 0.27)	0.52 (0.51, 0.54)	0.92 (0.90, 0.94)	1.34 (1.31, 1.36)	1.42 (1.40, 1.45)	70 (69, 72)
20 years			0.01 (0.01, 0.01)	0.04 (0.03, 0.04)	0.11 (0.10, 0.12)	0.26 (0.25, 0.27)	0.52 (0.50, 0.53)	0.92 (0.90, 0.94)	1.34 (1.31, 1.36)	1.42 (1.39, 1.45)	70 (69, 72)
30 years				0.03 (0.03, 0.03)	0.10 (0.09, 0.11)	0.25 (0.24, 0.26)	0.51 (0.50, 0.52)	0.91 (0.89, 0.93)	1.33 (1.30, 1.35)	1.41 (1.39, 1.44)	71 (69, 72)
40 years					0.07 (0.07, 0.08)	0.22 (0.21, 0.23)	0.48 (0.47, 0.50)	0.88 (0.87, 0.90)	1.31 (1.28, 1.33)	1.39 (1.37, 1.42)	72 (71, 73)
50 years						0.15 (0.14, 0.16)	0.41 (0.40, 0.43)	0.82 (0.80, 0.84)	1.25 (1.22, 1.27)	1.33 (1.31, 1.36)	75 (74, 77)
60 years							0.27 (0.26, 0.28)	0.68 (0.67, 0.70)	1.12 (1.10, 1.14)	1.21 (1.18, 1.23)	83 (81, 85)
70 years								0.43 (0.42, 0.45)	0.89 (0.87, 0.91)	0.98 (0.96, 1.00)	102 (100, 104)
80 years									0.52 (0.50, 0.53)	0.62 (0.60, 0.64)	161 (156, 166)
90 years										0.17 (0.16, 0.19)	278 (535, 625)

Values are presented as incidence (95% confidence interval) in percentages, except for the column “1 in:,” which shows numbers (95% confidence interval)

*ESKD* end-stage kidney disease

^a^“Eventually” corresponds to follow-up of more than 95 years

## Discussion

This study estimated the lifetime risk of ESKD in Japan by sex, revealing that the risk is higher in men than in women of equal age; approximately 1 in 32 men and 1 in 71 women in Japan possibly develop ESKD that results in maintenance dialysis therapy in their lifetime. We also determined age-conditional probabilities of developing ESKD by sex. These easily understandable statistics will be useful for assisting in public health education and planning in Japan.

Our study showed considerable sex-dependent differences in the lifetime risk of ESKD in Japan. This is in line with previous studies from other countries [12–14] that have found that men have higher lifetime risks of ESKD than women. Using cohort data of adults residing in Alberta, Canada, the lifetime risk of ESKD among those without ESKD at age 40 years was estimated to be 2.66% (95% CI 2.57–2.75%) for men and 1.76% (95% CI 1.69–1.83%) for women [12]. Using European Renal Association-European Dialysis and Transplant Association (ERA-EDTA) Registry data, the cumulative lifetime risk of ESKD up to age 90 years in Europe was estimated to be 1.40% or 0.73% for 40-year-old men or women, 1.32% or 0.68% for 50-year-old men or women, and 1.18% or 0.58% for 60-year-old men or women, respectively [13]. Using data from the United States Renal Data System (USRDS), the cumulative lifetime risk of ESKD from birth to age 100 + years in the total US population was estimated to be 3.96% (95% CI 3.93–3.99%) for men and 2.84% (95% CI 2.69–2.74%) for women [14]. Although simple comparisons cannot be made due to differences in methods and age categories used in each study, reported lifetime risk estimates of ESKD are less than 4% worldwide and are higher in men than in women. One possible explanation for the sex-dependent difference would be the higher incidence of ESKD in men than in women. According to the 2018 USRDS annual data report [15], the incidence of treated ESKD cases was substantially higher among males than among females in almost every country. The sex-dependent differences in the incidence of treated ESKD cases are thought to be due to, not only differences in the rate of chronic kidney disease (CKD) progression, but also differences in non-biological factors, such as personal preference and access to care [16].

Notably, our estimated lifetime risk of ESKD in Japan was lower than that reported for the United States [14]; these data were obtained using the same life-table methods of Fay [9] and Fay et al. [10] with DevCan software developed at the National Cancer Institute [11] (notably, the upper age range was 100 + years in the US study [14] whereas it was 95 + years in the present study). One might wonder why lifetime risk of ESKD in Japan was lower than that of the United States, given that the prevalence of treated ESKD is higher in Japan [15, 17]. While prevalence is influenced by both the rate at which new cases are emerging and the average duration of the disease, lifetime risk is influenced by the rate at which new cases are emerging but not the average duration of the disease. Although the prevalence of treated ESKD in Japan is higher than that in the United States, the incidence of treated ESKD in Japan is lower than that in the United States [15]. This explains why the lower estimated lifetime risk of ESKD in Japan than in the United States [14].

Compared to cumulative lifetime risks of other diseases in Japan, the estimated lifetime risk of ESKD was much lower. For example, the lifetime probability of developing cancer in 2001 was estimated to be 49.01% for men and 37.36% for women. These probabilities mean that 1 in 2 men and 1 in 3 women will develop cancer during their lifetime [18]. The cumulative lifetime incidence risk of gastric cancer was estimated to be 11.4% (1 in 9 people) for men and 5.7% (1 in 18 people) for women [19]. As for cardiovascular diseases, lifetime risk estimates of acute myocardial infarction at age 45 years, adjusted for competing risk of death, were 16.24% for men and 11.60% for women, based on data from a cohort of adults residing in Suita city, Japan (the Suita study) [20]. Using the same data, the lifetime risk of stroke at age 45 years, adjusted for competing risk of death, was estimated to be 18.93% for men and 20.18% for women [21].

Of course, a simple comparison between ESKD and other diseases is not feasible, and the meaning of lifetime risk values differs between ESKD and other diseases such as cancer and cardiovascular diseases. Some patients with cancer or cardiovascular disease, for example, may achieve cure, whereas there is no cure for ESKD. In other words, many people with ESKD live long lives while on dialysis or after having a kidney transplant without ever achieving a complete cure.

There are several limitations that need to be considered to adequately interpret our results. First, we defined an incident case of ESKD as a patient with loss of kidney function that resulted in maintenance dialysis therapy. Thus, only patients who had undergone dialysis treatment were included, as data were not available for patients who had not initiated dialysis or had undergone pre-emptive kidney transplantation in Japan. However, the number of patients who had not initiated dialysis were not included in the studies from other countries [12–14]. Furthermore, the number of pre-emptive kidney transplant patients is small in Japan, with only 101 patients being reported in 2009 [6]. Second, we assumed that both the number of incident cases of ESKD and the number of ESKD deaths for zero-year-olds were zero because these data were not available. However, given that both the number of incident cases of ESKD and the number of ESKD deaths for the 0–4 age group were very small, this assumption is unlikely to have had a major impact on our estimates. Finally, lifetime risk estimates are based on the assumption that exposure to risk- and age-specific rates remains stable. Regarding individual risk prediction, a limitation of lifetime risk estimates is that they do not take into account individual behaviors and risk factors, but are based on population-based data. For individual risk, the estimated lifetime risk will vary according to the individual’s remaining life expectancy, risk factor profile, and risk factor management success over time.

Despite these limitations, to the best of our knowledge, this study is the first to report the lifetime risk and age-conditional probability of ESKD in Japan. As the data were extracted from a nationwide survey of Japanese dialysis facilities and a national census, our findings should be broadly generalizable to the Japanese population. Furthermore, the method used in this study is easy to use for estimating cumulative incidence based on survey data. Enhanced awareness of these issues would enable nephrologists and healthcare professionals to advocate for the need to prevent the development and progression of CKD.

## Conclusions

The present study revealed that the cumulative incidences of ESKD from birth until age 95 years in Japan were 3.14% (95% CI 3.10–3.18%) and 1.42% (95% CI 1.39–1.44%) for men and women, respectively. These probabilities illustrate that approximately 1 in 32 men and 1 in 71 women in Japan will develop ESKD that results in maintenance dialysis therapy in their lifetime. This easily understandable information can be used to assist in public health education and planning.
